# Integrated proteomics of FFPE tissues reveals that upregulation of GALNT2 and SEC24A across cervical disease stages correlates with aggressive phenotype and poor prognosis

**DOI:** 10.1186/s12885-026-16745-1

**Published:** 2026-08-13

**Authors:** Qi Xie, Jingjing Wei, Yanling Zhang, Muyi Zhang, Kang Han, Shimeng Liu, Hongxiao Wang, Yuxue Zhang, Xiaoya Sun

**Affiliations:** 1 Department of Pathology, Henan Provincial People’s Hospital, People’s Hospital of Zhengzhou University, No. 7, Weiwu Road, Jinshui District, Zhengzhou, Henan China; 2 Department of Pathology, The Third Affiliated Hospital of Zhengzhou University, Zhengzhou, China; 3 Department of Outpatient Hysteroscopy Diagnosis and Treatment, The Fifth Affiliated Hospital of Zhengzhou University, No. 3 Kangfu Qianjie, Erqi District, Zhengzhou, Henan China

**Keywords:** Cervical cancer, Proteomics, FFPE, Metabolic reprogramming, Immune microenvironment, GALNT2, SEC24A

## Abstract

**Objective:**

The objective of this investigation is to elucidate the molecular mechanisms underlying cervical carcinogenesis, encompassing the transition from precancerous lesions to invasive carcinoma, through the analysis of proteomic alterations and the identification of key driver proteins.

**Methods:**

A comprehensive high-throughput proteomic analysis was conducted on 66 Formalin-Fixed, Paraffin-Embedded (FFPE) tissue samples, which included normal cervix (NC), high-grade squamous intraepithelial lesion (HSIL), and cervical cancer (CC). The employed bioinformatics methodologies included differential expression analysis, unsupervised clustering, Weighted Gene Co-expression Network Analysis (WGCNA), and multi-criteria screening based on consistent upregulation, prognostic relevance, module co-expression, and corroboration from existing literature. Validation was performed utilizing immunohistochemistry and external transcriptomic data from the TCGA-CESC cohort (*n* = 304).

**Results:**

The analysis successfully identified over 9,000 proteins, revealing significant heterogeneity among the NC, HSIL, and CC groups. The differential expression analysis demonstrated a notable increase in glycolytic metabolism alongside a decrease in oxidative phosphorylation as the disease progresses from NC to HSIL and then to CC, coupled with dynamic reprogramming of immune-related pathways, including the complement cascade. Unsupervised clustering identified three distinct molecular subtypes, each characterized by unique metabolic and immune microenvironmental features. These subtypes were independently validated in the TCGA-CESC transcriptomic cohort, which recapitulated the prognostic gradient and confirmed the progressive upregulation of GALNT2 across the subtypes. WGCNA identified modules significantly associated with clinicopathological parameters, such as tumor size, differentiation, and invasion depth. Through integrated screening, GALNT2 and SEC24A were identified as critical candidate driver proteins; their elevated expression was correlated with poorer patient survival, with immunohistochemistry and transcriptomic data confirming their progressive upregulation throughout the carcinogenic process.

**Conclusion:**

This study offers a comprehensive proteomic overview of cervical cancer progression and identifies GALNT2 and SEC24A as crucial regulators of metabolic-immune interactions, thereby providing novel mechanistic insights and potential therapeutic targets for cervical cancer.

**Supplementary Information:**

The online version contains supplementary material available at https://doi.org/10.1186/s12885-026-16745-1.

## Introduction

Cervical cancer, primarily driven by persistent high-risk human papillomavirus (HPV) infections, continues to pose a significant global health challenge despite advancements in screening and vaccination strategies [1, 2]. The transition from precancerous conditions, such as high-grade squamous intraepithelial lesions (HSIL), to invasive cervical carcinoma (CC) involves complex interactions among viral oncoproteins, accumulating genetic alterations within the host, and a dynamically evolving tumor microenvironment [3]. There remains a critical knowledge gap regarding the systematic protein-level regulatory networks that govern this malignant progression, which hampers the development of effective early diagnostic markers and targeted therapeutic strategies.

Proteomics has emerged as a critical approach for elucidating the functional determinants within cancer biology. While most studies predominantly utilize fresh-frozen tissues, the extensive collections of Formalin-Fixed, Paraffin-Embedded (FFPE) specimens available in clinical pathology represent an invaluable yet underutilized resource for clinically annotated retrospective research [4–6]. Recent technological advancements have enabled robust proteomic profiling of FFPE materials, thus offering direct insights into the molecular signatures preserved in routine clinical samples [7, 8]. This progress presents unprecedented opportunities to correlate molecular findings with long-term patient outcomes.Recent advancements in technology have facilitated robust proteomic profiling of FFPE materials, thereby providing a direct insight into the molecular signatures preserved within routine clinical samples. This advancement offers unprecedented opportunities to correlate molecular findings with long-term patient outcomes. A fundamental characteristic of cancer is the complex interaction between metabolic reprogramming and the remodeling of the immune microenvironment–a phenomenon referred to as “metabolic-immune crosstalk” [9, 10]. In the context of cervical cancer, HPV oncoproteins are recognized for disrupting host cell metabolism and fostering an immunosuppressive environment [11]. Nonetheless, a thorough and stage-specific understanding of the evolution of this metabolic-immune axis during progressive carcinogenesis, along with its primary molecular drivers, remains largely uncharted.

To bridge these knowledge gaps, we undertook an integrated proteomic investigation utilizing FFPE tissues that encompass the continuum from normal cervical tissue to high-grade squamous intraepithelial lesions (HSIL) and invasive cervical carcinomas (CC). Our objectives were threefold: (1) to delineate the global proteomic changes and pinpoint critical dysregulated pathways throughout cervical cancer progression; (2) to characterize the corresponding tumor microenvironment and categorize molecular subtypes based on intrinsic protein signatures; and (3) to implement a stringent, multi-step bioinformatic framework to identify and validate essential driver proteins with clinical significance. This study presents a comprehensive proteomic resource that clarifies the dynamic molecular landscape of cervical carcinogenesis, identifying GALNT2 and SEC24A as crucial regulators at the nexus of metabolism and immunity, thereby providing novel mechanistic and therapeutic insights into this disease.

## Materials and methods

### Patient samples and tissue preparation

A total of 66 formalin-fixed, paraffin-embedded (FFPE) tissue specimens were collected, comprising 20 normal cervical (NC) tissues, 22 high-grade squamous intraepithelial lesions (HSIL), and 24 invasive cervical carcinomas (CC). The healthy individuals in the normal cervix (NC) control group were age-matched to the patients in the HSIL and CC cohorts. For the HSIL group, samples were obtained from diagnostic biopsies (directed punch biopsies or LEEP/conization specimens); for the CC group, tissues were derived from definitive surgical resections. All patients were treatment-naïve prior to sample collection. All samples were obtained with informed consent and the study was approved by the Institutional Review Board. HPV detection and genotyping was performed on exfoliated cervical cells using PCR-based reverse-dot blot hybridization. Serial sections from all clinical samples were reviewed by a pathologist to estimate the percentages of neoplastic/lesional cells relative to adjacent normal epithelial or stromal tissue. The estimated ranges are as follows: for the normal cervix (NC) group, 85–95% epithelial cells (with minimal stromal contamination); for the HSIL group, 60–85% lesional cells; and for the CC group, 50–80% tumor cells. This histological assessment was used to account for tissue heterogeneity in subsequent protein expression analyses. For proteomic analysis, ten consecutive 10-µm thick sections were cut from each FFPE block. Deparaffinization and protein extraction were performed using the established FFPE tissue proteomics protocol, involving heat-induced antigen retrieval in lysis buffer containing SDS and protease inhibitors [12].

### Protein extraction and digestion

FFPE tissue Sect.  (10 μm thick) were prepared using the FFomic strategy. H&E-guided sections were deparaffinized with xylene and washed through an ethanol gradient. Under a dissecting microscope, specimens were scraped and lysed in TCEP buffer (2% deoxycholic acid sodium salt, 40 mM 2-chloroacetamide, 100 mM Tris-HCl, 10 mM Tris(2-chloroethyl) phosphate, 1 mM PMSF, pH 8.5) supplemented with protease and phosphatase inhibitors at 99 °C for 30 min. After cooling to room temperature, trypsin was added and digestion was performed at 37 °C for 18 h. Peptides were extracted with 0.1% formic acid in 50% acetonitrile and dried using a SpeedVac.

### LC-MS/MS analysis

Peptides were analyzed using an OE480 Hybrid Quadrupole‑Orbitrap MS (Thermo Fisher) coupled with an EASY‑nLC 1200. Dried peptides were dissolved in Solvent A (0.1% formic acid in water), loaded onto a 2‑cm trap column (100 μm i.d.), and separated on a 15‑cm column (150 μm i.d., 1.9 μm C18‑AQ beads) at 600 nL/min over a 120‑min gradient: 0–8 min, 4–15% B; 8–100 min, 15–35% B; 100–110 min, 35–50% B; 110–111 min, 50–100% B; 111–120 min, 100% B (Solvent B: 0.1% formic acid in 80% ACN). Eluted peptides were ionized at 2.4 kV. MS data were acquired in DDA mode. MS1: Orbitrap at 120,000 resolution, m/z 300–1400, AGC 3 × 10⁶, max IT 80 ms. MS2: ion trap rapid mode, HCD NCE 27%, AGC 5 × 10⁴, max IT 20 ms, dynamic exclusion 12 s [13].

### MS acquisition

Data-dependent acquisition (DDA) mode with Top 20 method. MS1 full scan: m/z 300–1,400, resolution 120,000, AGC target 3 × 10⁶, maximal ion injection time 80 ms. MS2: rapid scan mode, HCD fragmentation with normalized collision energy 30%, resolution 7,500, AGC target 5 × 10⁴, maximal ion injection time 22 ms, dynamic exclusion 40 s. FAIMS compensation voltages: −45V and − 65V.” (Revised Methods section) [14].

### Data quality control and preprocessing

The iProteome one-stop cloud platform (qualitative+quantitative). UniProt human database.The quality of the proteomic data was rigorously assessed. Proteins identified in less than 70% of samples within any group were removed. The LFQ intensities were log2-transformed, and missing values were imputed using the k-nearest neighbors (KNN) algorithm from the impute R package. Principal component analysis (PCA) was performed to evaluate the overall data structure and sample clustering using the FactoMineR version 2.8 package [15].

### Differential expression and pathway enrichment analysis

Differential protein expression analysis between groups (HSIL vs. NC, CC vs. HSIL, CC vs. NC) was conducted using the limma package in R. Proteins with an absolute log2 fold change > 0.585 (~ 1.5-fold) and an adjusted p-value (Benjamini-Hochberg) < 0.05 were considered significantly differentially expressed. Functional enrichment analysis of Gene Ontology (GO) biological processes and Kyoto Encyclopedia of Genes and Genomes (KEGG) pathways [16] was performed for the up- and down-regulated protein sets using the clusterProfiler package, with a significance threshold of adjusted p-value < 0.05.

### Gene set variation analysis (GSVA) and immune microenvironment assessment

To investigate the activity of specific biological pathways across different disease stages (HSSL, early-CC, late-CC), Gene Set Variation Analysis (GSVA) was performed using the GSVA package with the “ssgsea” method. The “c2.cp.kegg.v2023.1” gene set collection from MSigDB was used [17]. The cellular composition of the tumor microenvironment was estimated using the xCell algorithm based on the protein expression data to infer immune and stromal cell type abundances [18].

## Unsupervised molecular subtyping

Consensus clustering was applied to the protein expression matrix of all samples using the ConsensusClusterPlus package1.62.0 [19]. The analysis was performed with Pearson correlation distance and the partitioning around medoids (PAM) algorithm, with 80% resampling over 1000 iterations. The optimal number of clusters (k) was determined by evaluating the cumulative distribution function (CDF) and the relative increase in the area under the CDF curve. The resulting subtypes were characterized by their distinct pathway activities and immune microenvironment features.

### Weighted gene co-expression network analysis (WGCNA)

A weighted gene co‑expression network was built using the WGCNA R package (v1.72‑1). The input was a FOT intensity matrix of 7,011 proteins detected in ≥ 50% of samples. Because the DDA data have complex missingness, we first assessed missing patterns (MAR and MNAR) via group comparison and Little’s MCAR test. To enable downstream analysis, missing values were imputed using K‑nearest neighbors (k = 10, Euclidean distance), a method suitable for DDA proteomics that minimally disturbs original correlations (Pearson change < 0.05 in simulations). The imputed matrix was used for module construction.

A soft‑thresholding power β = 6 was chosen based on scale‑free topology. A signed adjacency matrix (Pearson correlation) was built and converted to a topological overlap matrix (TOM) [20]. Dynamic tree cutting (height cut = 40,000) identified 21 modules (including the grey module). Module eigengenes (first principal component of each module) were correlated with clinical traits (tumor diameter, differentiation grade, invasion depth) using Pearson correlation, with Bonferroni correction. Ordinal variables (differentiation: well = 1, moderate = 2, poor = 3) were numerically encoded. Spearman rank correlation was used as a sensitivity analysis for all module‑trait associations.

### Bioinformatic screening and co-expression network analysis

Candidate proteins were initially selected by analyzing proteomic data from NC, HSIL, and CC tissues. proteins that were consistently upregulated during disease progression and significantly associated with poor patient prognosis were identified (14 proteins). Subsequently, Weighted Gene Co-expression Network Analysis (WGCNA) was applied to further prioritize proteins within modules strongly correlated with aggressive clinical phenotypes, such as tumor diameter and invasion depth. This step refined the list to 9 core candidate proteins enriched in these malignancy-associated modules [21]. Literature Curation and Prognostic Validation: A systematic literature review was conducted for the shortlisted proteins to prioritize them based on existing evidence of their roles in oncoproteinsis. Focus was placed on proteins with well-documented pro-tumorigenic functions (GALNT2 and SEC24A). Their prognostic value was then validated using survival data from public cohorts, confirming that high expression of GALNT2 and SEC24A was significantly associated with shorter overall survival, establishing them as top candidate drivers. For consensus clustering subtypes, survival was compared across the three molecular subtypes (ccp1, ccp2, ccp3). Kaplan-Meier survival analysis with log-rank test was performed proteins.

### Histological evaluation and immunohistochemistry (IHC)

To validate the proteomic findings at the tissue level, we first performed hematoxylin-eosin (HE) staining on an independent set of FFPE tissue sections, including samples of normal cervix (NC), high-grade squamous intraepithelial lesion (HSIL), and cervical carcinoma (CC), for histological assessment and pathological grading. Subsequently, immunohistochemical staining was carried out as follows: after deparaffinization and antigen retrieval, the sections were incubated with primary antibodies specific for GALNT2 (Cat No. 17441-1-AP) and SEC24A (Cat No. 15958-1-AP). Appropriate positive and negative controls were included in each experiment. Signal detection was achieved using a DAB substrate, followed by hematoxylin counterstaining. Two pathologists, blinded to the sample groups, independently evaluated the histological features under HE staining as well as the IHC staining intensity and percentage of positive cells. A semi-quantitative H-score was then calculated for each sample to objectively assess the expression levels of the target proteins across different stages of disease progression.

### Validation in the TCGA-CESC transcriptomic cohort

TCGA‑CESC RNA‑Seq data (GENCODE v36, TPM) for 304 primary cervical tumors were obtained from the GDC, and clinicopathological annotations were retrieved from cBioPortal. Consensus clustering (ConsensusClusterPlus) was performed on the top 3,000 most variable genes (median absolute deviation among genes significantly different across NC, HSIL, and CC in the proteomics cohort; Kruskal–Wallis FDR < 0.05) using Pearson correlation distance, 1,000 iterations of 80% subsampling, and average‑linkage hierarchical clustering (k = 3, fixed seed = 42). Overall survival was compared by log‑rank test; FIGO stage and keratinization were assessed by Chi‑square or Fisher’s exact tests (two‑sided). Subtype‑specific differentially expressed genes were identified by Kruskal–Wallis (P < 0.01) with ΔZ filtering (C0‑Inter > 1.0; C1‑Late and C2‑Early > 0.5). Pathway enrichment was performed with Enrichr (KEGG 2021 Human), and Hallmark pathway activity was scored as mean per‑gene Z‑scores (MSigDB Hallmark v2026.1), with differential activity tested by Kruskal–Wallis (P < 0.05) and ΔZ filtering.

### Statistical analysis

All statistical analyses were performed using R software (version 4.2.0). Continuous variables were compared using Student’s t-test or Wilcoxon rank-sum test, as appropriate. Categorical variables were compared using the Chi-square test or Fisher’s exact test. Correlation analyses were performed using Pearson’s or Spearman’s method. A two-sided p-value < 0.05 was considered statistically significant unless otherwise specified for multiple testing corrections. Differential expression analysis was performed using the empirical Bayes moderated t-test (limma package). Multiple testing correction was applied using the Benjamini-Hochberg false discovery rate (FDR) method. Significance threshold: Differentially expressed proteins (DEPs) were defined as those with an absolute log₂fold change > 0.585 (corresponding to a fold change of approximately 1.5, since log₂1.5 ≈ 0.585) and an adjusted P-value (Benjamini-Hochberg FDR) < 0.05. In the Results section and figures, all reported P-values are FDR-adjusted P-values. All source data tables include both raw p-values and FDR-adjusted P-values.” (Revised Methods section).

Data representation: (1) Main quantitative analyses (differential expression, WGCNA, survival): FOT (Fraction of Total) intensities; (2) Heatmaps for visualization: Z-score transformation (row-wise) of FOT values, which facilitates comparison of expression patterns across proteins with different absolute abundances; (3) Boxplots and scatter plots: FOT intensities (original scale); (4) Differential expression: fold change (FC) and log₂(FC) calculated from group mean FOT values. The term log₁₀(FOT) has been replaced with FOT throughout.

## Results

### Quality control of proteomics data

**Fig. 1:**
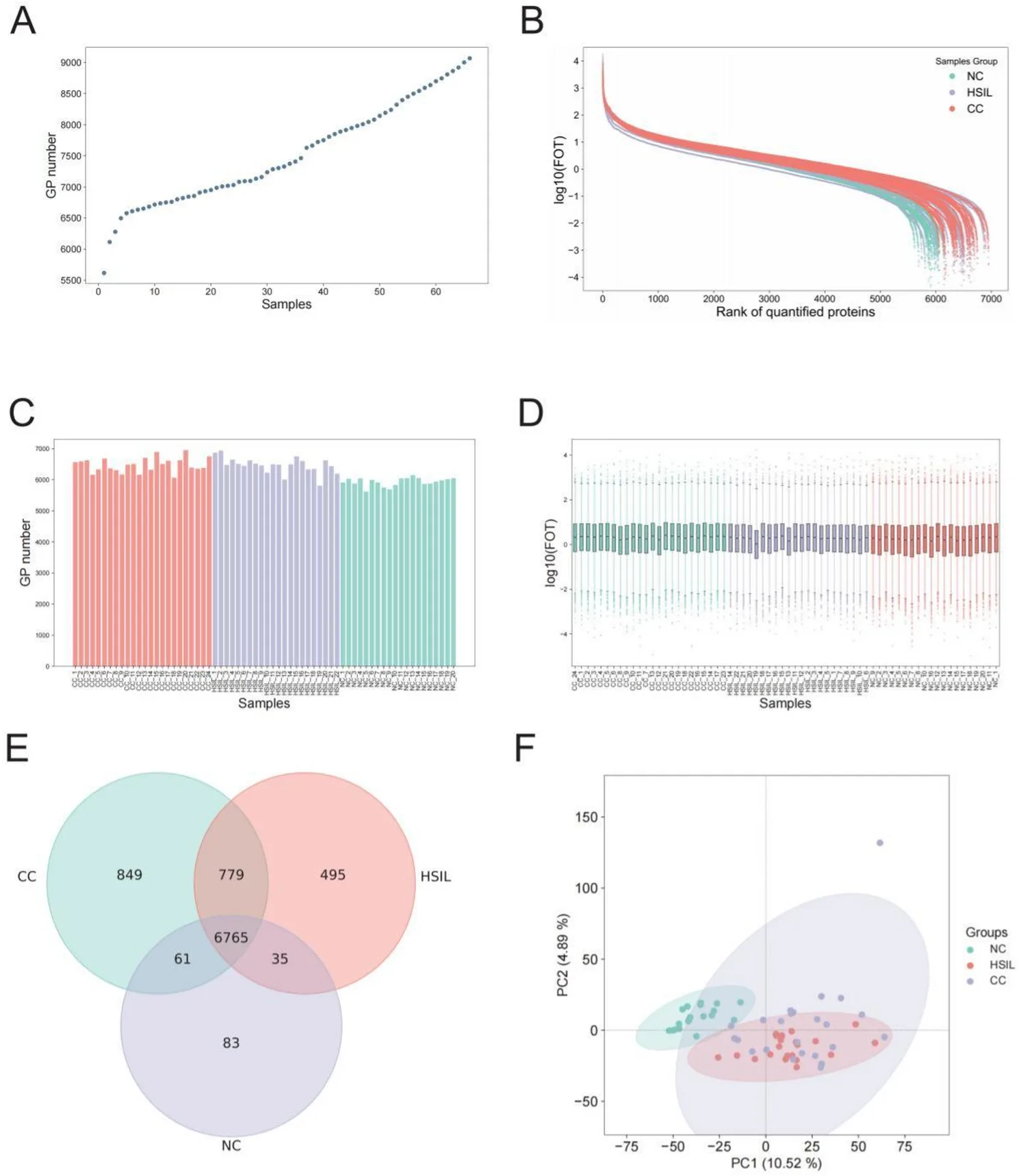
Quality control and overview of the proteomic landscape across cervical tissues. **A** Scatter plot showing the total number of proteins identified in the study. **B** Protein abundance distribution curves for individual samples across normal cervix (NC), high-grade squamous intraepithelial lesion (HSIL), and cervical cancer (CC) groups. **C** Box plot displaying the number of proteins reliably detected in each sample. **D** Box plot illustrating the median protein expression intensity across samples, spanning eight orders of magnitude. **E** Venn diagram depicting the overlap of expressed proteins among the NC, HSIL, and CC groups. **F** Principal component analysis (PCA) plot demonstrating clear separation of NC samples from HSIL and CC samples based on global proteomic profiles

We conducted stringent quality control procedures on all samples. Proteomic profiling successfully identified more than 9,000 proteins (Fig. 1A), reflecting comprehensive coverage of the proteome. The expression distribution of proteins across individual samples is illustrated in Fig. 1B, where each curve corresponds to one sample, the vertical axis denotes expression intensity, and the horizontal axis represents the rank order of protein abundance. Notably, marked differences in protein expression were evident among the three groups. Although the distinctions between the CC and HSIL groups were subtle, the NC group displayed considerable divergence in protein expression relative to both disease groups (Fig. 1B).

As depicted in Fig. 1C, over 6,000 proteins were consistently detected in each sample, indicating robust proteomic depth. Median expression levels were comparable across samples, and protein abundances ranged across eight orders of magnitude (Fig. 1D), confirming effective detection of both high- and low-abundance proteins without notable quantitative distortion. Subsequent quantitative evaluation of histones identified 6,765 proteins commonly expressed across all three groups. Interestingly, the number of proteins uniquely expressed in the NC group was relatively limited (approximately 80 proteins; Supplementary Table S1, Fig. 1E). Principal component analysis further underscored substantial overall distinctions among the groups, particularly between the NC group and the two pathological groups (CC and HSIL; Fig. 1F). Supplementary Table S2 lists the top 100 differentially expressed proteins (DEPs) for each comparison, ranked by ascending FDR. All reported P-values were adjusted using the Benjamini-Hochberg FDR method.

### Analysis of differential expression between HSIL and NC groups

**Fig. 2:**
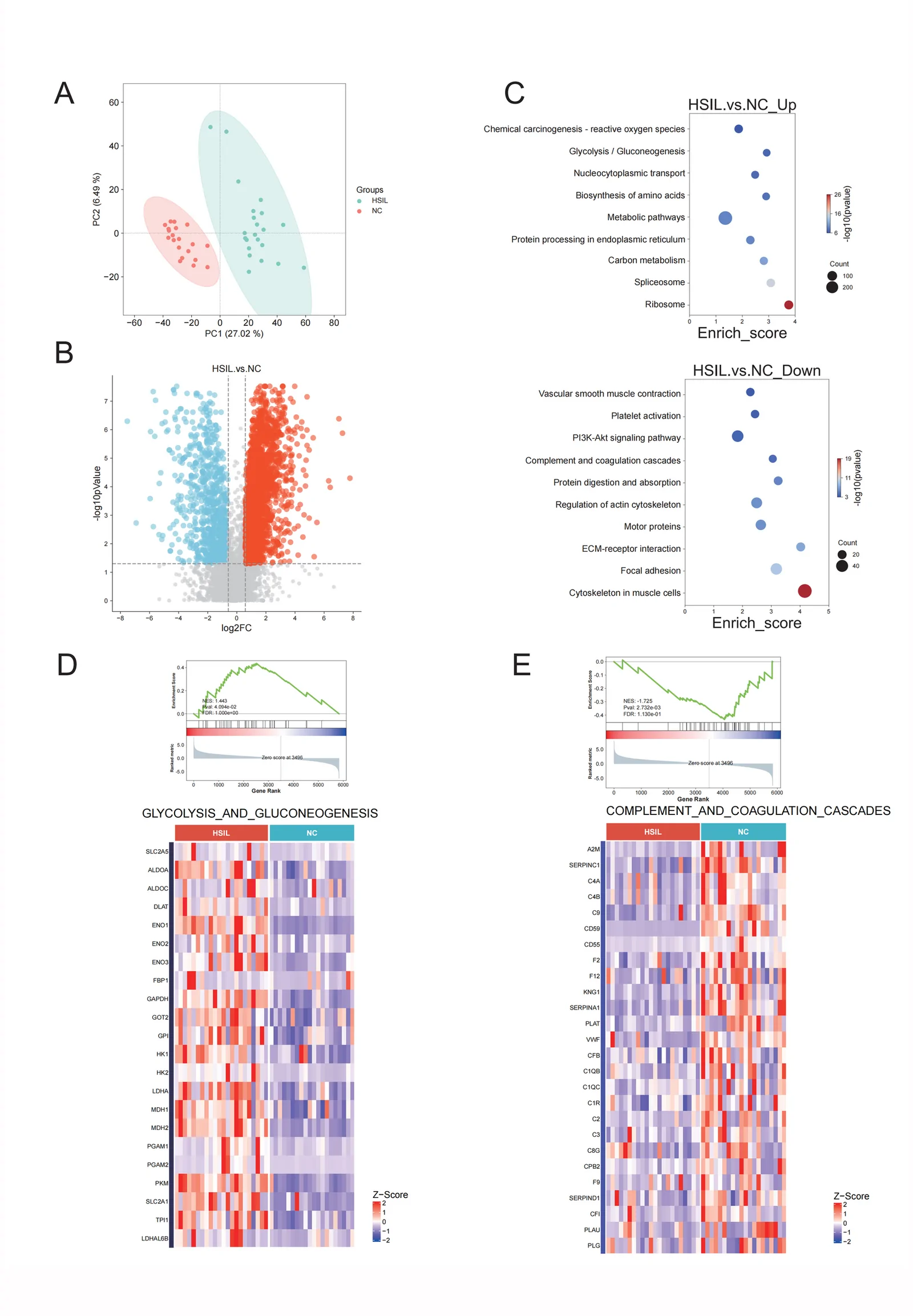
Proteomic alterations between HSIL and normal cervical tissues. **A** PCA plot showing greater heterogeneity within the HSIL group compared to the tightly clustered NC group. **B** Volcano plot identifying proteins differentially expressed between HSIL and NC tissues. **C** Bar plot summarizing the significantly upregulated biological pathways in HSIL versus NC. **D** Schematic representation and expression trends of key molecules involved in the upregulated pathways in HSIL, including glycolytic enzymes (e.g., DLAT, LDHA) and transporters (SLC2A1). **E** Key complement/coagulation proteins upregulated in NC and downregulated in HSIL

Principal component analysis (PCA) of the proteomic data revealed that samples from the NC (normal control) group were relatively tightly clustered, whereas those from the HSIL (high-grade squamous intraepithelial lesion) group exhibited a more dispersed distribution. This pattern indicates substantial heterogeneity among HSIL patients (Fig. 2A). Furthermore, volcano plot analysis identified a large number of proteins with significantly differential expression between the two groups (Fig. 2B).Specifically, compared to the NC group, upregulated proteins in the HSIL group were primarily enriched in reactive oxygen species (ROS)-related signaling pathways, suggesting the presence of oxidative stress in these patients. Additionally, elevated activity was observed in glycolytic processes and amino acid metabolism. In contrast, proteins upregulated in the NC group were mainly associated with complement activation, extracellular matrix organization, and cytoskeleton-related signaling pathways. At a systemic level, both the complement system and coagulation cascade were activated in healthy individuals (Fig. 2C). As visually summarized in Fig. 2D, representative upregulated pathways and their molecular expression trends in the HSIL group are illustrated. Key enzymes involved in glycolysis and gluconeogenesis (such as DLAT and LDHA) were significantly elevated. The rate-limiting enzymes HK1 and HK2, as well as the transporter SLC2A1, also showed increased expression. In NC, complement-related proteins including C4A and C4B were upregulated, along with several immune markers and coagulation-associated proteins such as F2 and F12. Moreover, elevated expression was detected for immune-related proteins CD55 and CD59 (Fig. 2E).

### Differential analysis results between CC and HSIL

**Fig. 3:**
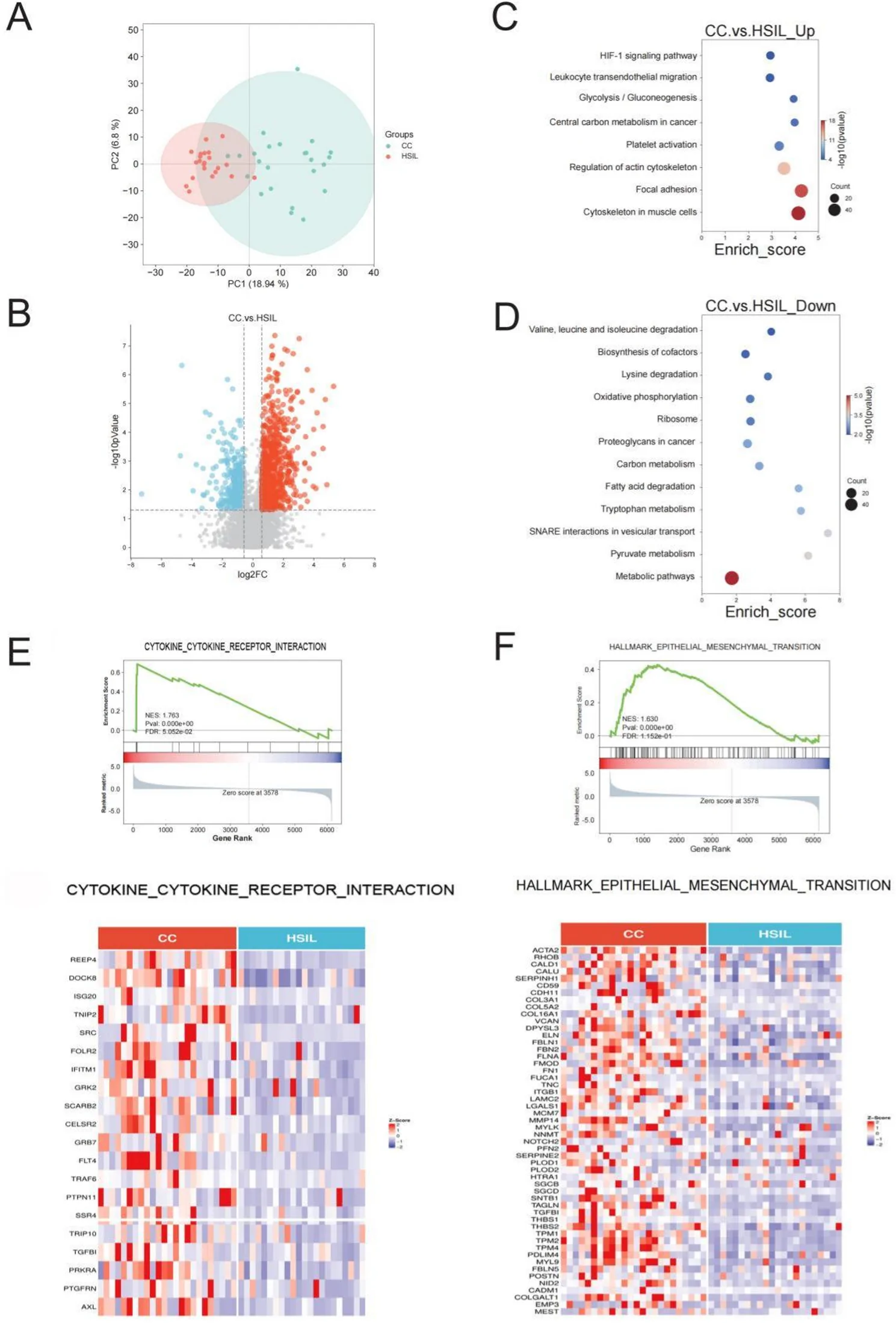
Molecular differences between cervical cancer and HSIL. **A** PCA plot indicating partial separation between the CC and HSIL groups. **B** Volcano plot of differentially expressed proteins between CC and HSIL. **C** Pathway enrichment analysis highlighting biological processes upregulated in CC compared to HSIL. **D** Comparative activity of glycolysis and oxidative phosphorylation pathways in CC and HSIL. **E**, **F** Expression patterns of key receptor tyrosine kinases **E** and epithelial-mesenchymal transition (EMT)-related molecules **F** in CC versus HSIL

Principal component analysis (PCA) revealed a partial separation between the CC and HSIL groups, indicating distinguishable but overlapping molecular profiles (Fig. 3A). We identified a set of differentially expressed proteins between these groups (Fig. 3B), prompting further investigation into their associated signaling pathways.Pathway analysis showed significant upregulation in CC of glycolysis, cytoskeleton reorganization, amino acid and fatty acid metabolism, and oxidative phosphorylation (Fig. 3C). Notably, we observed stage-specific enrichment in glucose metabolism: glycolysis was elevated in CC, whereas oxidative phosphorylation was more active in HSIL (Fig. 3D). Although less efficient in ATP production, glycolysis supports tumor proliferation by generating essential biosynthetic intermediates.Coagulation pathways were also upregulated in CC, displaying dynamic changes during progression. Key receptor tyrosine kinases-including FLT1, FLT4, KDR, and PDGFRA-showed elevated expression in CC (Fig. 3E). Furthermore, epithelial-mesenchymal transition (EMT) was activated in CC tissues, accompanied by increased expression of collagens and MMPs (Fig. 3F), suggesting enhanced invasive potential and aggressive tumor behavior.

### Differential expression and pathway enrichment analysis between CC and NC tissues

**Fig. 4:**
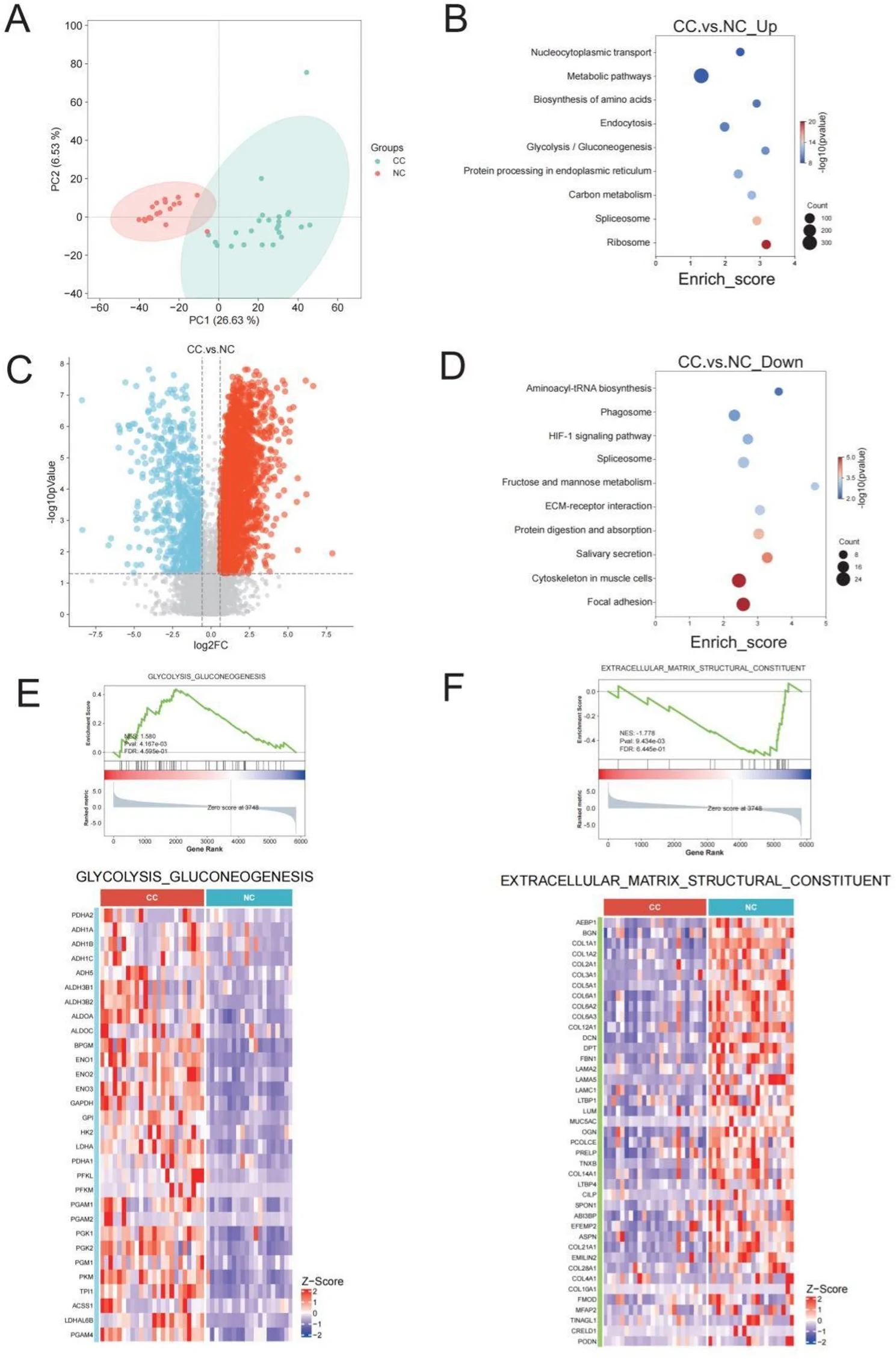
Comparative proteomic profiling of cervical cancer and normal tissues. **A** PCA plot showing pronounced separation between CC and NC groups. **B** Enrichment analysis indicating significant upregulation of the glycolytic pathway (Warburg effect) in CC. **C** Volcano plot of differentially expressed proteins between CC and NC. **D** Pathways upregulated in NC tissues compared to CC. **E** Expression levels of key Warburg effect-related glycolytic enzymes in CC. **F** Enrichment of stromal and extracellular matrix (ECM) components in NC tissues

A comparative analysis was conducted between CC and NC tissues. Principal component analysis revealed pronounced separation between the two groups, indicating significant global proteomic differences (Fig. 4A).Further investigation demonstrated significant upregulation of the glycolytic signaling pathway in the CC group, which is consistent with the Warburg effect (Fig. 4B). Volcano plot analysis confirmed a substantial number of differentially expressed proteins (DEPs) between CC and NC groups (Fig. 4C). Volcano plot analysis identified 2,907 DEPs in HSIL vs. NC (2,025 up-regulated, 882 down-regulated), 1,145 DEPs in CC vs. HSIL (804 up-regulated, 341 down-regulated), and 2,971 DEPs in CC vs. NC (2,277 up-regulated, 694 down-regulated) (Fig. 4A-C, Supplementary Table S3).

In contrast, pathways upregulated in NC tissues were primarily associated with alternative fructose metabolic pathways, cytoskeletal organization, and extracellular matrix (ECM) components (Fig. 4D).Notably, the CC group exhibited elevated expression of key glycolytic enzymes related to the Warburg effect (Fig. 4E). Interestingly, NC tissues showed marked enrichment of various stromal components (Fig. 4F). Compared to our previous findings in high-grade squamous intraepithelial lesions (HSIL), CC tissues demonstrated higher expression of multiple stromal components, including various collagens. Furthermore, matrix metalloproteinases (MMPs), which facilitate ECM degradation, were also elevated in CC tissues, suggesting enhanced invasive potential. In contrast, NC tissues primarily showed increased collagen expression without concomitant MMP upregulation, reflecting a normal stromal phenotype without invasive characteristics.

To further explore upstream regulatory networks, we performed GSVA-based upstream regulator analysis using MSigDB Hallmark and transcription factor (TF) motif sets. This revealed 31 significantly enriched Hallmark pathways and 367 TF motifs (FDR < 0.05) in CC versus NC. Key findings include upregulation in CC of pathways linked to metabolic reprogramming (e.g., PI3K-AKT-mTOR signaling, glycolysis, MYC targets) and in NC of pathways related to epithelial-mesenchymal transition (EMT), WNT signaling, and angiogenesis. Distinct TF motifs (e.g., CEBPB/TCF11MAFG enriched in CC; CDPCR3/PAX3 enriched in NC) further underscore divergent regulatory landscapes. These results provide a data-driven, experimentally supported overview of protein-level regulatory networks in malignant progression (Supplementary Figure S1).

### Analysis of differential disease progression

For disease progression analysis, samples were subclassified into three groups representing tumor progression (Supplementary Table S4): H1 represents HSIL (n = 22), CC1 represents well-differentiated FIGO stage IA-IB1 (n = 14), and CC2 represents poorly-differentiated FIGO stage IB2-IIA (n = 10), with differentiation grading following the WHO Classification of Tumours of Female Reproductive Organs (5th edition). Molecular analysis revealed a gradual reduction in early and late estrogen response across these groups, along with high DNA repair activity specifically in CC1. Additionally, multiple pathways—including complement signaling, hypoxia, interferon-alpha response, PI3K-AKT-mTOR signaling, epithelial-mesenchymal transition, and angiogenesis–were activated in both CC1 and CC2, whereas KRAS signaling inhibitors were significantly upregulated in H1.

**Fig. 5:**
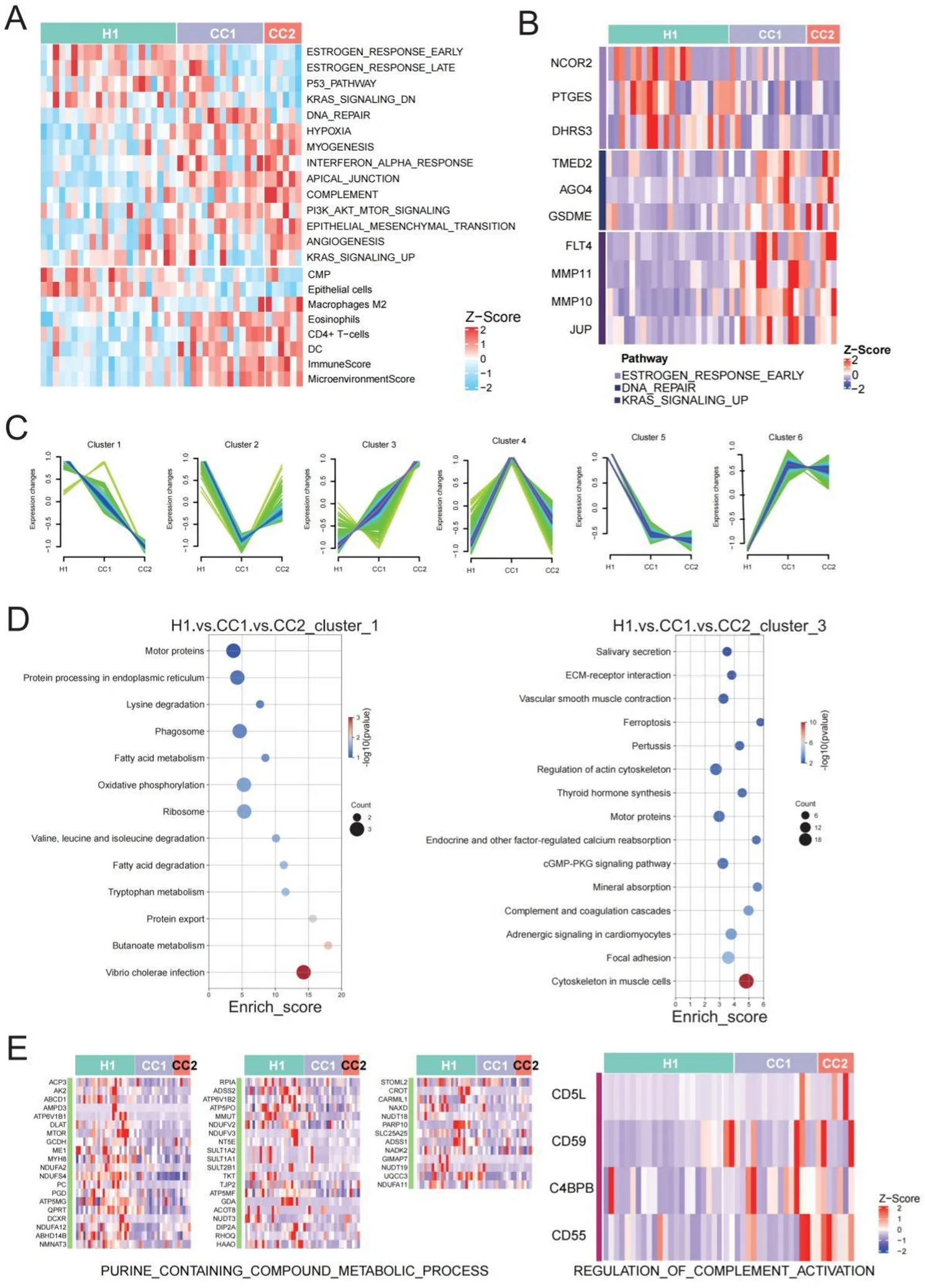
Dynamic molecular and immune microenvironment remodeling during disease progression. **A** Evaluation of immune and microenvironment scores across HSIL (H1, *n* = 22), well-differentiated carcinoma (CC1, *n* = 14), and poorly-differentiated carcinoma (CC2, *n* = 10) groups. **B** Z-score heatmap displaying the expression patterns of representative proteins across the progression groups. **C** Patterns of progressively downregulated and upregulated proteins. **D** Correlation of key pathways (e.g., oxidative phosphorylation, ECM-related processes) with malignancy. **E** Stage-specific expression patterns of proteins involved in purine metabolism and complement regulation

From the tumor microenvironment perspective, epithelial cells were highly expressed in HSIL, while macrophages, CD4 + T cells, and dendritic cells were elevated in CC1 and CC2, with M2 macrophages particularly enriched in CC2. Immune and microenvironment scores were higher in carcinoma groups, indicating enhanced immunosuppression and immune infiltration (Fig. 5A). Gene expression analysis via Z-score heatmap (Fig. 5B) revealed decreased expression of estrogen-responsive proteins (e.g., NCOR2, PTGES) from H1 to CC2, elevated DNA repair proteins (e.g., TMED2, GSDME) in CC1, and upregulation of complement, hypoxia, interferon-alpha, and angiogenesis-related proteins (e.g., FLT4, MMP11) in CC1 and CC2. The progressive downregulation and upregulation patterns may suggest potential tumor suppressor or putative oncoproteins associations, respectively (Fig. 5C). Key pathways such as oxidative phosphorylation (negatively correlated with malignancy) and ECM-related processes (positively correlated) were identified (Fig. 5D). Purine metabolism and complement regulatory proteins (e.g., CD59, CD55) exhibited stage-specific expression, with some proteins peaking in CC1 or CC2, indicating metabolic reprogramming and immune microenvironment remodeling during progression (Fig. 5E).In summary, these results highlight dynamic molecular and immune changes associated with tumor malignant progression.

### Proteomics-based molecular subtyping reveals distinct disease heterogeneity

**Fig. 6:**
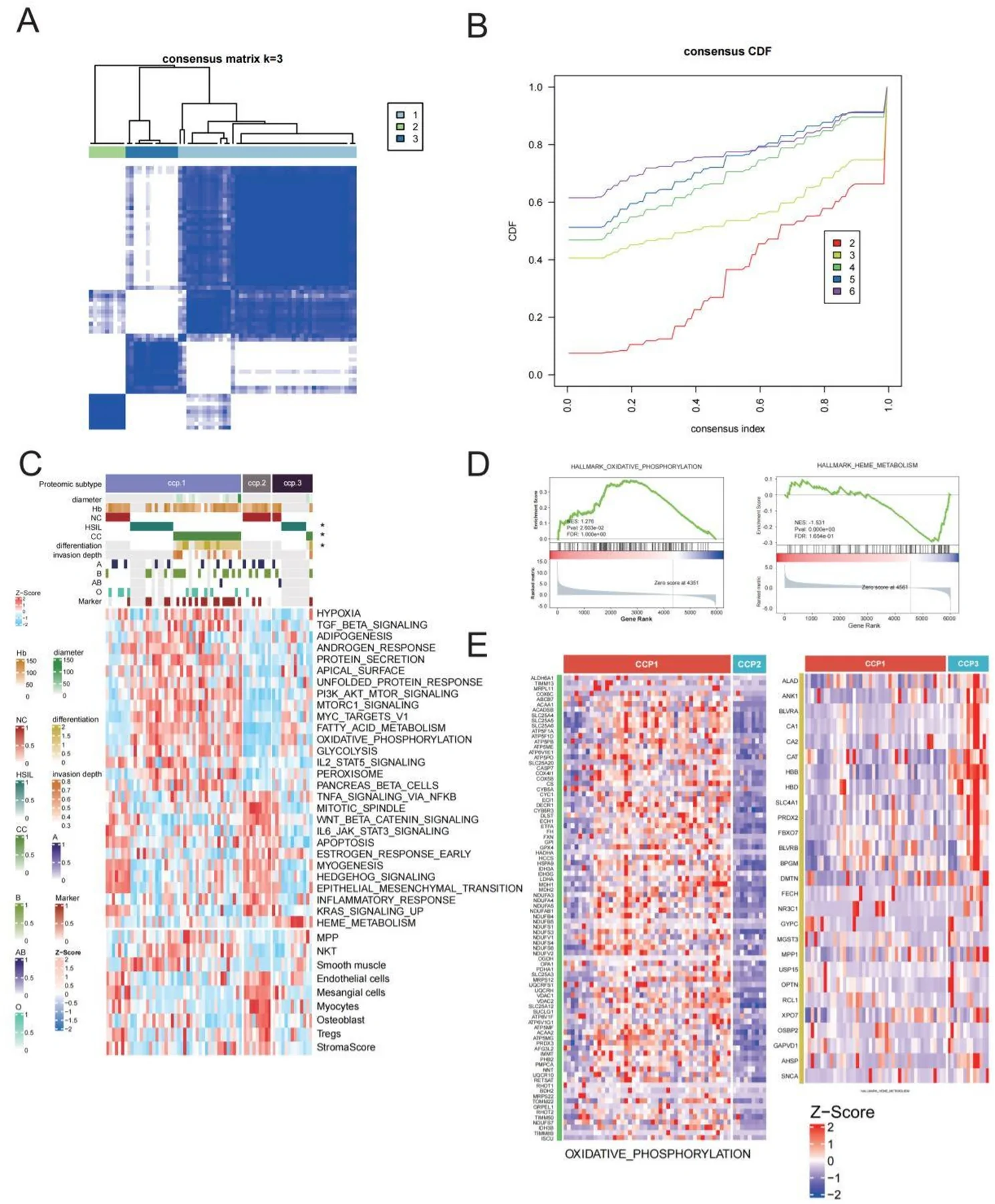
Proteomics-based molecular subtyping reveals intrinsic disease heterogeneity. **A** Consensus clustering matrix $\:\mathrm{k}=3$ for all samples. **B** Cumulative distribution function (CDF) plot determining the optimal cluster number $\:\mathrm{k}=3$. **C** Heatmap and annotation summarizing the defining molecular features, activated pathways, and tumor microenvironment characteristics of the three subtypes (ccp1, ccp2, ccp3). **D**, **E** Gene Set Enrichment Analysis (GSEA) plots validating subtype-specific pathway activities, including oxidative phosphorylation in ccp1 (**D**) and downregulated heme metabolism in ccp3 (**E**)

Given the substantial heterogeneity among patients with identical pathological stages, we performed unsupervised consensus clustering on proteomic data to delineate intrinsic molecular subtypes within the control and disease cohorts (Fig. 6A). The optimal and most stable classification was achieved with three subtypes, as indicated by the greatest increase in the cumulative distribution function (CDF) (Fig. 6B).Subtype characterization revealed distinct molecular and microenvironmental features (Fig. 6C). The ccp1 subtype, predominantly CC cases, was defined by activated PI3K-AKT and IL2-STAT5 signaling, along with fatty acid and glycolysis metabolism. Its tumor microenvironment was marked by NKT cell infiltration. In contrast, the ccp2 subtype consisted exclusively of NC, featuring WNT signaling, estrogen response, and epithelial-mesenchymal transition (EMT) pathways, with a stromal cell-rich microenvironment. The ccp3 subtype, largely composed of HSIL samples, was characterized by heme metabolism—a process associated with antioxidant functions that may aid tumor cells in countering oxidative stress and promoting angiogenesis. Notably, patients with poorly differentiated or deeply invasive tumors were primarily classified into the ccp1 subtype.

Gene set enrichment analysis (GSEA) further validated the pathway activities specific to each subtype (Fig. 6D, E). The ccp1 subtype showed significant enrichment in oxidative phosphorylation * NES=1.276,P=0.026*, indicating active energy metabolism. Conversely, the ccp3 subtype exhibited marked downregulation in heme metabolism * NES=-1.531,P<0.001*, corroborating its metabolic dysfunction in this pathway. These findings underscore profound metabolic reprogramming differences among molecular subtypes, providing novel insights into the functional basis of disease heterogeneity.

### Identification of clinically relevant co-expression modules via WGCNA

**Fig. 7:**
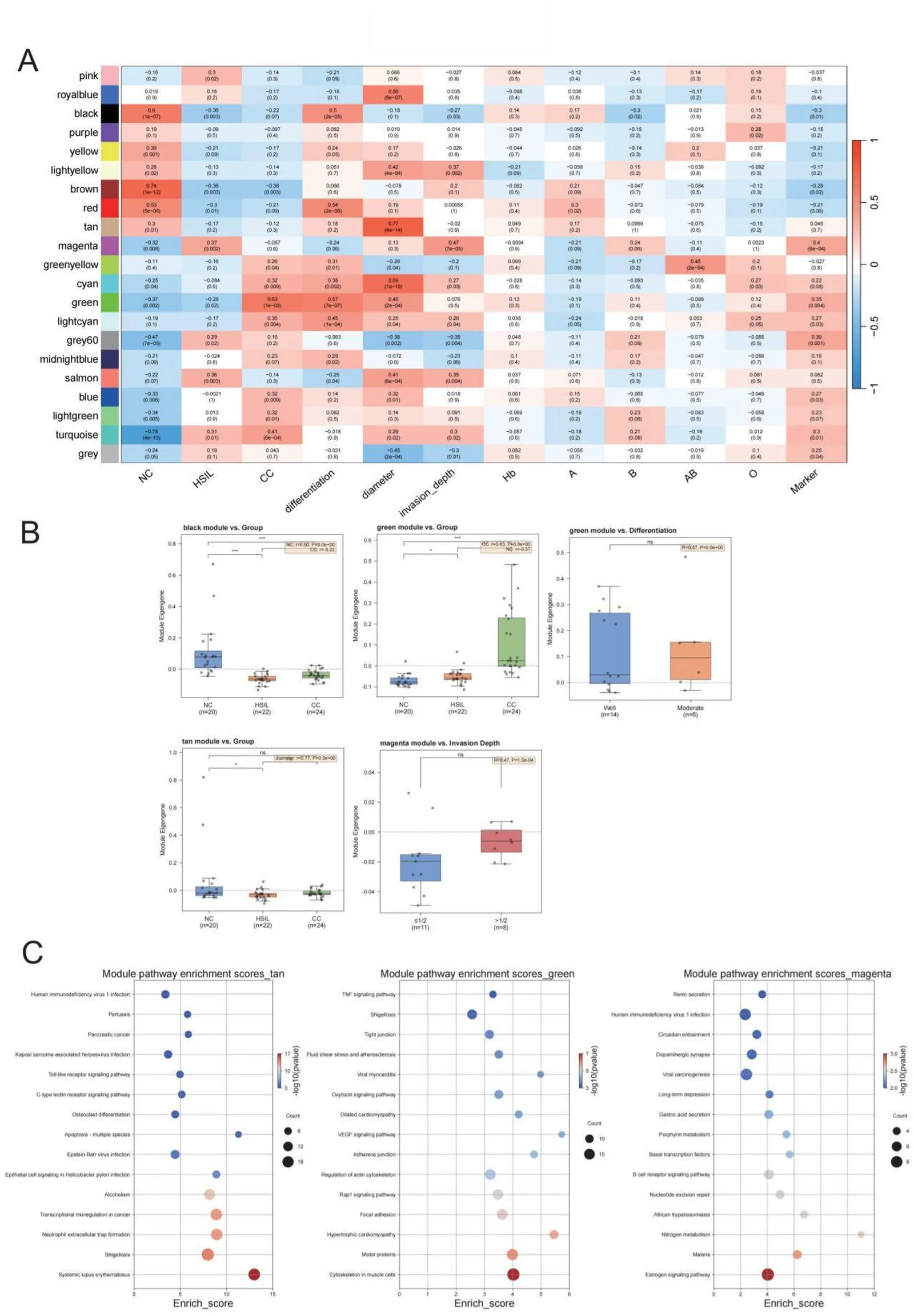
Identification of clinically relevant co-expression modules via WGCNA. **A** Hierarchical clustering dendrogram of proteins and identified co-expression modules. **B** The tumor diameter scatter plot has been retained (continuous variable) with a loess smoothing curve in addition to the linear fit. **C** Bar plot displaying the enrichment scores of representative biological pathways within the tan, green, and magenta modules

We performed weighted gene co-expression network analysis (WGCNA) to identify protein co-expression modules and assess their correlations with key clinicopathological features. A total of 20 modules (excluding the grey module for unassigned proteins, n = 894) were identified, with module sizes ranging from 92 (royalblue) to 1,773 (turquoise) proteins (Fig. 7A). Scatter plots for categorical/ordinal traits (differentiation, invasion depth) have been replaced with boxplots stratified by category, showing the distribution of module eigengene values for each category (e.g., well-differentiated, moderately differentiated, poorly differentiated), with individual data points overlaid (Fig. 7B).

Additionally, the black module (n = 301) was positively correlated with normal tissue (R = 0.60). Enrichment scores for key pathways in each module are detailed in Fig. 7C, and the complete list of proteins with module membership (kME) values is provided in Supplementary Table S5. Collectively, these findings link specific co-expression modules to distinct clinical phenotypes, highlighting roles for immune activation, cytoskeletal dynamics, and metabolic reprogramming in tumor growth, differentiation, and aggressiveness.

### Multi-step screening and experimental validation of key driver proteins in cervical cancer

**Fig. 8:**
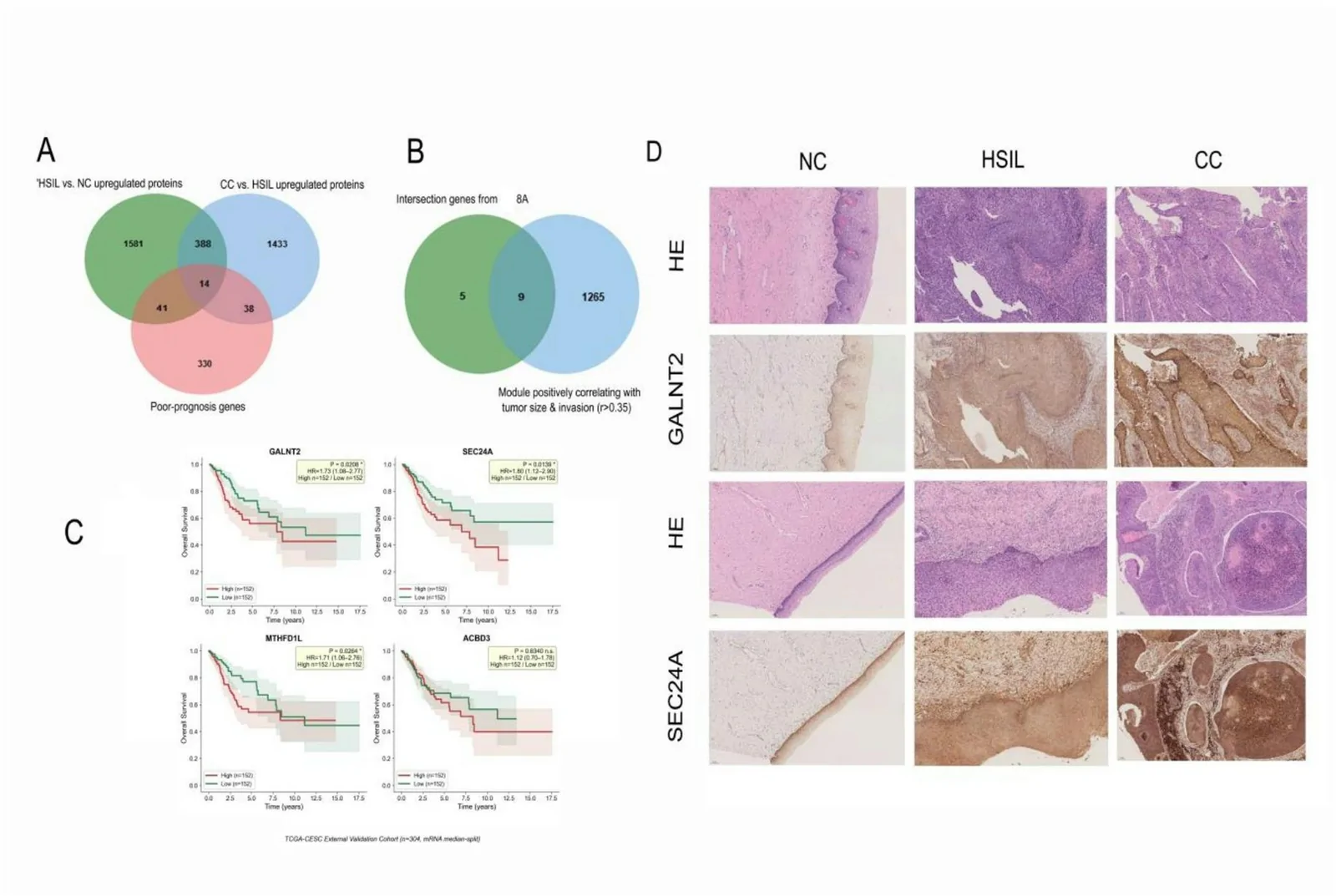
Multi-step screening and validation of key driver proteins in cervical cancer. **A** Venn diagram intersecting proteins consistently upregulated from NC to HSIL to CC with proteins associated with poor prognosis, yielding 14 initial candidates. **B** Intersection of the 14 candidates with proteins from WGCNA modules positively correlated with tumor diameter and invasion depth, narrowing the list to 9 proteins. **C** TCGA-CESC KM survival (*n* = 304). GALNT2 (*P* = 0.021) and SEC24A (*P* = 0.014) high expression = worse OS. **D** Immuno-histochemistry (IHC) validation showing progressive upregulation of GALNT2 and SEC24A protein expression from NC to HSIL to CC tissues

To systematically identify core driver proteins in cervical carcinogenesis and progression, we established a rigorous multi-step bioinformatic screening pipeline. Initially, by comparing normal cervical (NC), high-grade squamous intraepithelial lesion (HSIL), and cervical cancer (CC) samples, we identified candidate proteins that were consistently up-regulated during lesion progression and closely associated with poor prognosis. This preliminary analysis yielded 14 candidate proteins (Fig. 8A). Subsequently, weighted gene co-expression network analysis (WGCNA) was applied to identify gene modules positively correlated with tumor diameter and invasiveness (correlation coefficient > 0.35). Intersection of these module proteins with the initial candidate proteins revealed nine proteins–DIAPH2, GALNT2, MYBBP1A, SEC24A, MTHFD1L, ACBD3, VPS33A, COG3, and SH3PXD2B—that were significantly enriched in malignancy-related modules, suggesting their potential key roles in tumor growth and invasion (Fig. 8B).

We further evaluated the known functions of these nine proteins through a systematic literature review. Existing evidence indicated that GALNT2, SEC24A have been widely reported as putative oncoproteins in multiple cancer types, whereas MYBBP1A has been primarily characterized as a tumor suppressor. The remaining proteins showed unclear associations with tumorigenesis. Based on the stronger oncogenic evidence for GALNT2 and SEC24A, we focused subsequent prognostic analyses on these two proteins, with the patient distribution defined as follows: for GALNT2, a high-expression group (n = 12) and a low-expression group (n = 12) were determined by a median split of FOT intensity in CC samples; for SEC24A, the same median-based split yielded high-expression (n = 12) and low-expression (n = 12) groups; additionally, the subtype distribution included ccp1 (n = 28, events = 11), ccp2 (n = 20, events = 0), and ccp3 (n = 18, events = 4), with detailed per-sample data, including expression values and clinical characteristics, provided in Supplementary Table S6; Using the TCGA-CESC cohort (n = 304, 72 events), we performed de novo Kaplan-Meier survival analyses via the cBioPortal API with median‑based stratification, log‑rank tests, and Cox proportional hazards regression. High GALNT2 expression was significantly associated with worse overall survival (P = 0.021, HR = 1.73, 95% CI 1.08–2.77). Similarly, high SEC24A expression predicted a poorer prognosis (P = 0.014, HR = 1.80, 95% CI 1.12–2.90). In contrast, ACBD3 expression showed no significant prognostic value (P = 0.634, HR = 1.12, 95% CI 0.70–1.78) (Fig. 8C).

Finally, the independent validation cohort for the IHC phase consisted of 5 independent clinical samples per group, we validated the protein expression of GALNT2 and SEC24A using immunohistochemistry (IHC) in independent clinical samples. The results were highly consistent with proteomics data, showing a progressive up-regulation from normal cervical tissue through HSIL to cervical cancer (Fig. 8D). This multi-omics consistency reinforces the roles of GALNT2 and SEC24A as core drivers in cervical cancer development.

### External validation and transcriptomic characterization of CCP subtypes in TCGA-CESC

**Fig. 9:**
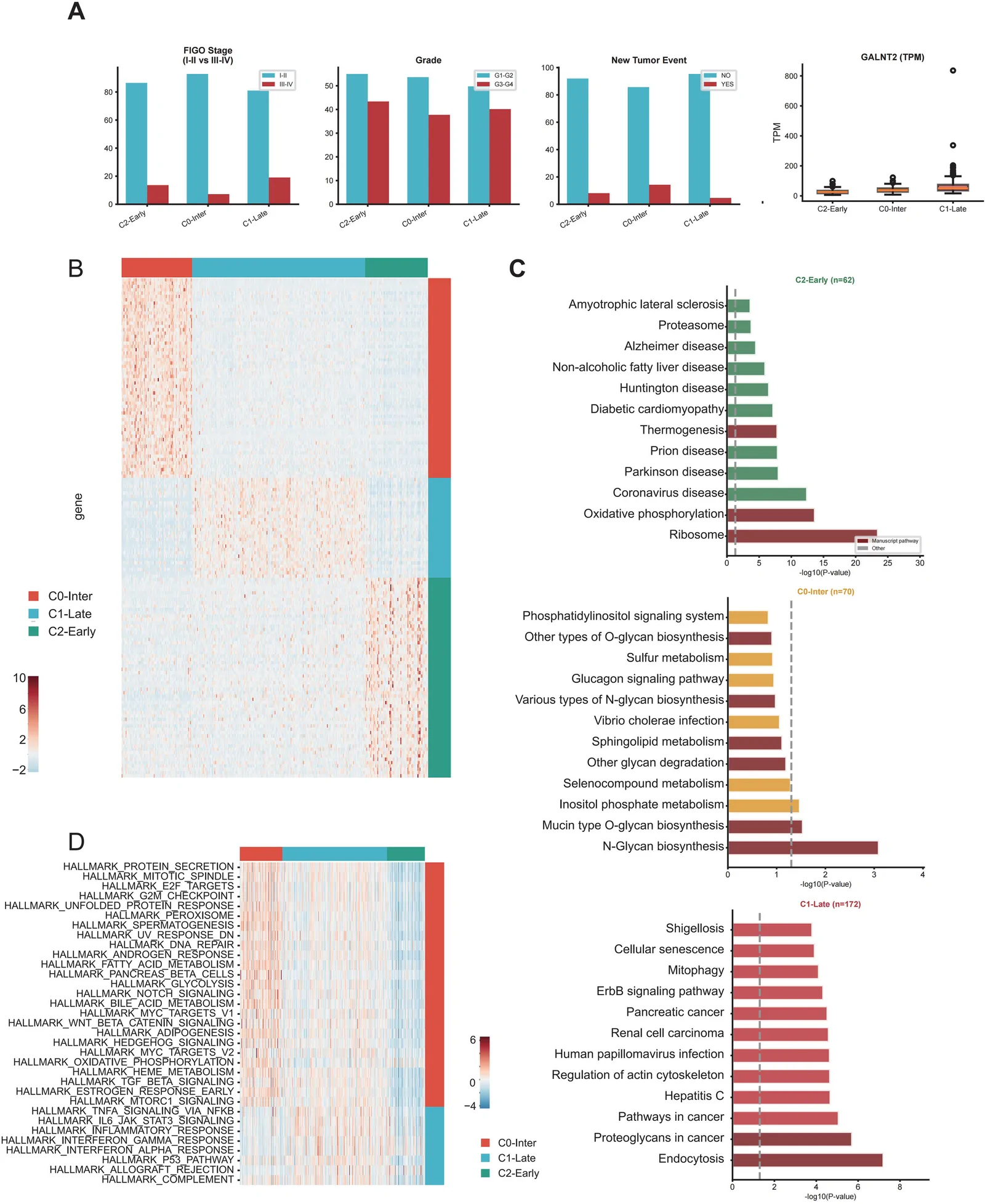
External validation of CCP molecular subtypes in TCGA-CESC. **A** Clinical and molecular features by subtype. Overall survival differed significantly (log-rank *P* = 0.0089). C1-Late was enriched for advanced FIGO stage (Fisher’s *P* = 0.042) and keratinizing SCC (Chi-square *P* = 0.012). GALNT2 mRNA increased monotonically (29.2→41.0→67.4 TPM). **B** Heatmap of top 60 subtype-specific upregulated genes per subtype (Kruskal–Wallis *P* < 0.01, ΔZ thresholds as indicated). **C** Top 12 KEGG pathways per subtype (Enrichr). C0-Inter: N-/O-glycan biosynthesis; C1-Late: endocytosis, actin regulation, cancer pathways. **D** Hallmark pathway activity (ssGSVA). C0-Inter: metabolic/proliferative programs; C1-Late: inflammatory/immune pathways (TNFα-NFκB, IL6-JAK-STAT3, interferon-γ, complement)

To validate the proteomics-derived subtyping framework, consensus clustering was applied to the TCGA-CESC transcriptomic dataset (*n* = 304), identifying three subtypes: C2-Early (*n* = 62), C0-Inter (*n* = 70), and C1-Late (*n* = 172). These subtypes recapitulated the internal prognostic gradient (overall survival log-rank *P* = 0.0089; event rates: 8.1%, 25.7%, 28.5%). C1-Late (the aggressive ccp1 counterpart) was enriched for FIGO stage III–IV tumors (28.5% vs. 18.2%, Fisher’s *P* = 0.042) and keratinizing squamous cell carcinoma (37.0% vs. 27.5%, χ² *P* = 0.012) (Fig. 9A). Consistent with the proteomics findings, GALNT2 mRNA expression increased monotonically across subtypes (29.2 → 41.0 → 67.4 TPM), confirming its progressive upregulation in cervical carcinogenesis (Figure A). Transcriptomic profiling identified large sets of subtype-specific differentially expressed genes, with the top 60 per subtype illustrated in a heatmap (Supplementary Table S7; Fig. 9B).

Pathway analysis further supported biological validity. C0-Inter was enriched in N-glycan and O-glycan biosynthesis (KEGG *P* = 6.0 × 10⁻⁴ and 2.5 × 10⁻²), linking to GALNT2 glycosyltransferase function, while C1-Late showed endocytosis, actin cytoskeleton remodeling, and cancer pathway activation (*P* = 1.9 × 10⁻⁷, 2.3 × 10⁻⁶, 6.7 × 10⁻⁶) (Fig. 9C). Hallmark analysis revealed a metabolic–inflammatory polarization: C0-Inter was dominated by metabolic reprogramming (oxidative phosphorylation, glycolysis, PI3K-AKT-mTOR, TGF-β signaling), whereas C1-Late was characterized by inflammatory signaling (TNFα-NFκB, IL6-JAK-STAT3, interferon-γ response, complement) (Fig. 9D). This duality mirrors the concurrent metabolic reprogramming and immune microenvironment remodeling described in the proteomics study.

## Discussion

This study delineates the molecular landscape of cervical carcinogenesis through integrated proteomic analysis of FFPE tissues. We charted dynamic alterations across the normal–HSIL–CC spectrum, characterized the immune microenvironment, defined molecular subtypes, and identified key drivers. The progressive activation of glycolysis (Warburg effect) and suppression of oxidative phosphorylation were accompanied by significant immune remodeling [22–24]. Carcinoma groups showed increased infiltration of macrophages, CD4 + T cells, and dendritic cells, with specific M2 macrophage enrichment in poorly differentiated tumors, indicating a shift toward an immunosuppressive milieu [25]. The co-activation of complement, hypoxia, and angiogenesis pathways underscores a central metabolic-immune axis in pathogenesis, where metabolic reprogramming directly fuels an environment conducive to immune evasion and tumor progression [26–28].

This aligns with a growing paradigm in oncology, where tumors are recognized not merely as masses of proliferating cells but as dysfunctional organs that corrupt local and systemic physiology [29]. We identified three molecular subtypes (ccp1–ccp3), revealing substantial disease heterogeneity beyond histological grading. The aggressive ccp1 subtype, defined by PI3K-AKT signaling and glycolytic metabolism, may be susceptible to targeted therapies like AKT inhibitors or drugs targeting the glycolytic pathway. Its association with NKT cell infiltration also presents a unique opportunity for immunotherapy strategies aimed at activating this lymphocyte population. The ccp3 subtype, predominantly HSIL, showed impaired heme metabolism, suggesting a premalignant adaptive mechanism that could inform interception strategies [30, 31]. This metabolic vulnerability, linked to oxidative stress management, might be exploited therapeutically using pro-oxidant agents to selectively target these high-risk precancerous lesions, a concept known as “ferroptosis induction” gaining traction in cancer prevention. The ccp2 subtype, consisting solely of NC with active WNT and estrogen signaling, provides a crucial molecular baseline from which disease-associated deviations can be accurately measured. Importantly, these proteomics-derived subtypes were independently validated in the TCGA-CESC transcriptomic cohort (n = 304), where consensus clustering recapitulated the prognostic gradient and confirmed the progressive upregulation of GALNT2 across subtypes, supporting the robustness and clinical relevance of the classification.

Weighted gene co-expression network analysis (WGCNA) linked specific protein modules to clinical phenotypes: the tan module with tumor diameter/immune pathways, the green module with poor differentiation/cytoskeletal reorganization, and the magenta module with invasion depth/nitrogen metabolism. These modules provide a functional framework for understanding aggressiveness and potential signatures for risk stratification [32–34]. For instance, the strong correlation between the tan module (enriched in systemic lupus erythematosus pathways) and tumor size suggests that chronic, dysregulated inflammatory states may paradoxically foster tumor expansion, possibly through creating a perpetually activated yet ineffective immune response [35]. The green module’s link to cytoskeletal dynamics underscores the fundamental role of cell shape, motility, and adhesion in determining differentiation status and metastatic potential. Meanwhile, the magenta module’s association with nitrogen metabolism and invasion depth hints at how tumors might rewire nutrient scavenging pathways to support invasion into deeper tissue structures. A core achievement is the multi-step validation of GALNT2 and SEC24A as key drivers. Both proteins showed consistent upregulation from NC to CC, association with malignant phenotypes, prognostic value for overall survival, and validation via IHC and external transcriptomic data. GALNT2, a glycosyltransferase, can modify cell surface receptors and immune interactions. Its role in other cancers often involves modulating receptor tyrosine kinase signaling and immune checkpoint molecule function [36]; for example, GALNT2-mediated glycosylation can stabilize PD-L1 on the cell surface [37–39]. SEC24A, part of the COPII complex, regulates protein secretion, potentially of immune modulators and MMPs [40]. Their roles likely bridge tumor-intrinsic functions (glycosylation, secretion) and microenvironment shaping, positioning them as potential therapeutic targets within the metabolic-immune axis [41, 42]. We hypothesize a synergistic model: SEC24A drives the hypersecretion of pro-tumorigenic factors (like MMPs for ECM remodeling or VEGF for angiogenesis), while GALNT2 modifies these factors or their receptors to optimize signaling and evasion from immune surveillance. This axis represents a promising but unexplored vulnerability in cervical cancer.

While the use of FFPE tissues enhances clinical relevance, potential protein artifacts or epitope masking from formalin fixation cannot be fully excluded, which may affect the detection of some low-abundance or particularly sensitive proteins. The cohort size, though well-annotated, may affect subtype generalizability, necessitating validation in larger, independent, and ideally multi-center cohorts to ensure robustness and clinical applicability. The functional roles of GALNT2 and SEC24A, particularly in metabolic-immune crosstalk, require mechanistic validation in vitro and in vivo.

Future work must move beyond correlation to establish causation. This includes: (1) Functional assays: Using CRISPR/Cas9 or RNAi to knock down GALNT2/SEC24A in cervical cancer cell lines to assess impacts on proliferation, invasion, glycolysis, and secretome profile. (2) Microenvironment modeling: Employing 3D co-culture systems with immune cells to study how tumor-cell-intrinsic ablation of these proteins alters immune cell recruitment, polarization, and cytotoxic activity. (3) In vivo validation: Testing the therapeutic effect of inhibiting these targets, alone or in combination with immune checkpoint blockade, in immunocompetent mouse models or patient-derived xenograft (PDX) models with humanized immune systems. (4) Translational exploration: Screening for existing or developing small-molecule inhibitors that could modulate the activity of these drivers.

### Limitations

Several limitations of this investigation warrant recognition. Primarily, although the utilization of FFPE specimens holds clinical significance, formalin-induced cross-linking may generate protein artifacts or obscure epitopes, thereby potentially compromising the identification of low-abundance or conformation-dependent proteins. Secondly, the cohort size (n = 66) remains relatively limited; despite comprehensive annotation, this constraint could restrict the broader applicability of the proposed molecular subtypes. While external validation using the TCGA-CESC transcriptomic dataset (n = 304) independently corroborated the prognostic gradient and the progressive elevation of GALNT2, offering cross-platform support, this verification is confined to mRNA levels. Consequently, it fails to fully replicate the proteomic dimension or the tumor-specific microdissection employed in the discovery phase. Furthermore, the protein signatures defining these subtypes, alongside the functional contributions of GALNT2 and SEC24A within metabolic-immune crosstalk, remain correlational in nature, necessitating mechanistic exploration through both in vitro and in vivo models. Lastly, although WGCNA modules associated with tumor dimensions, differentiation status, and invasion depth present promising risk indicators, these correlations lack validation in independent cohorts. Future endeavors should prioritize large-scale, multi-center proteomic studies that integrate orthogonal validation strategies and functional experiments.

## Conclusion

In summary, our multi-omics analysis proposes a coherent model where metabolic reprogramming and immune microenvironment remodeling are synergistically co-opted during cervical cancer progression, potentially driven by a nexus of molecules including GALNT2 and SEC24A. These findings advocate for a shift in clinical strategy–from a uniform approach to biomarker-driven subtyping and therapeutic interventions aimed at disrupting the critical metabolic-immune interface. The ccpl subtype, for instance, emerges as a clear candidate for metabolic pathway inhibitors. Ultimately, this work provides not just a descriptive molecular atlas but a functional roadmap, paving the way for developing more precise, mechanism-based combinations of metabolic, targeted, and immunotherapeutic agents to improve outcomes for patients across the spectrum of cervical neoplasia.

## Supplementary Information

Supplementary Material 1. Fig. S1. Upstream Regulator Analysis GSVA Hallmark Pathways and C3 TF Motifs (CC vs. NC). Two horizontal bar panels (A: Hallmark, B: C3 TF motifs) with CC-high (red) and NC-high (blue) color coding.

Supplementary Material 2.

Supplementary Material 3.

Supplementary Material 4.

Supplementary Material 5.

Supplementary Material 6.

Supplementary Material 7.

Supplementary Material 8.

## Data Availability

The datasets used and/or analyzed during the current study are available from the corresponding author on reasonable request.
